# Surgical treatment for symptomatic ventriculus terminalis: case series and a literature review

**DOI:** 10.1007/s00701-019-03996-0

**Published:** 2019-07-05

**Authors:** Alexander Fletcher-Sandersjöö, Erik Edström, Jiri Bartek, Adrian Elmi-Terander

**Affiliations:** 1 Department of Neurosurgery, Karolinska University Hospital, Solna, Sweden; 2 Department of Clinical Neuroscience, Karolinska Institutet, Bioclinicum J5:20, 171 64 Solna, Sweden; 3 Department of Neurosurgery, Rigshospitalet, Copenhagen, Denmark; 4 Department of Medicine, Karolinska Institutet, Stockholm, Sweden

**Keywords:** Ventriculus terminalis, Terminal ventricle of Krause, Fifth ventricle, Neurosurgery, Surgery, Conus medullaris

## Abstract

**Background:**

Ventriculus terminalis is a cystic embryological remnant within the conus medullaris that normally regresses after birth. In rare cases, it may persist into adulthood and give rise to neurological symptoms, for which the optimal treatment remains uncertain. The aim of this study was to present our experience from a population-based cohort of patients with ventriculus terminalis and discuss our management strategy as compared to the existing literature.

**Methods:**

A retrospective review was conducted of all adult (≥ 15 years) patients with ventriculus terminalis who were referred to the Karolinska University Hospital between 2010 and 2018.

**Results:**

Fourteen patients were included. All patients were symptomatic at the time of referral, and the most common symptom was lower limb weakness (*n* = 9). Microsurgical cyst fenestration was offered to all patients and performed in thirteen. Postoperative imaging confirmed cyst size reduction in all surgically treated patients. No surgical complications were reported. Eleven of the surgically treated patients showed clinical improvement at long-term follow-up. One patient declined surgery, with progression of the cyst size and clinical deterioration observed at follow-up.

**Conclusions:**

Surgery for ventriculus terminalis seems to be a safe and effective option for relief of symptoms. We propose that surgery should be offered to all patients with symptomatic ventriculus terminalis.

## Introduction

**Fig. 1:**
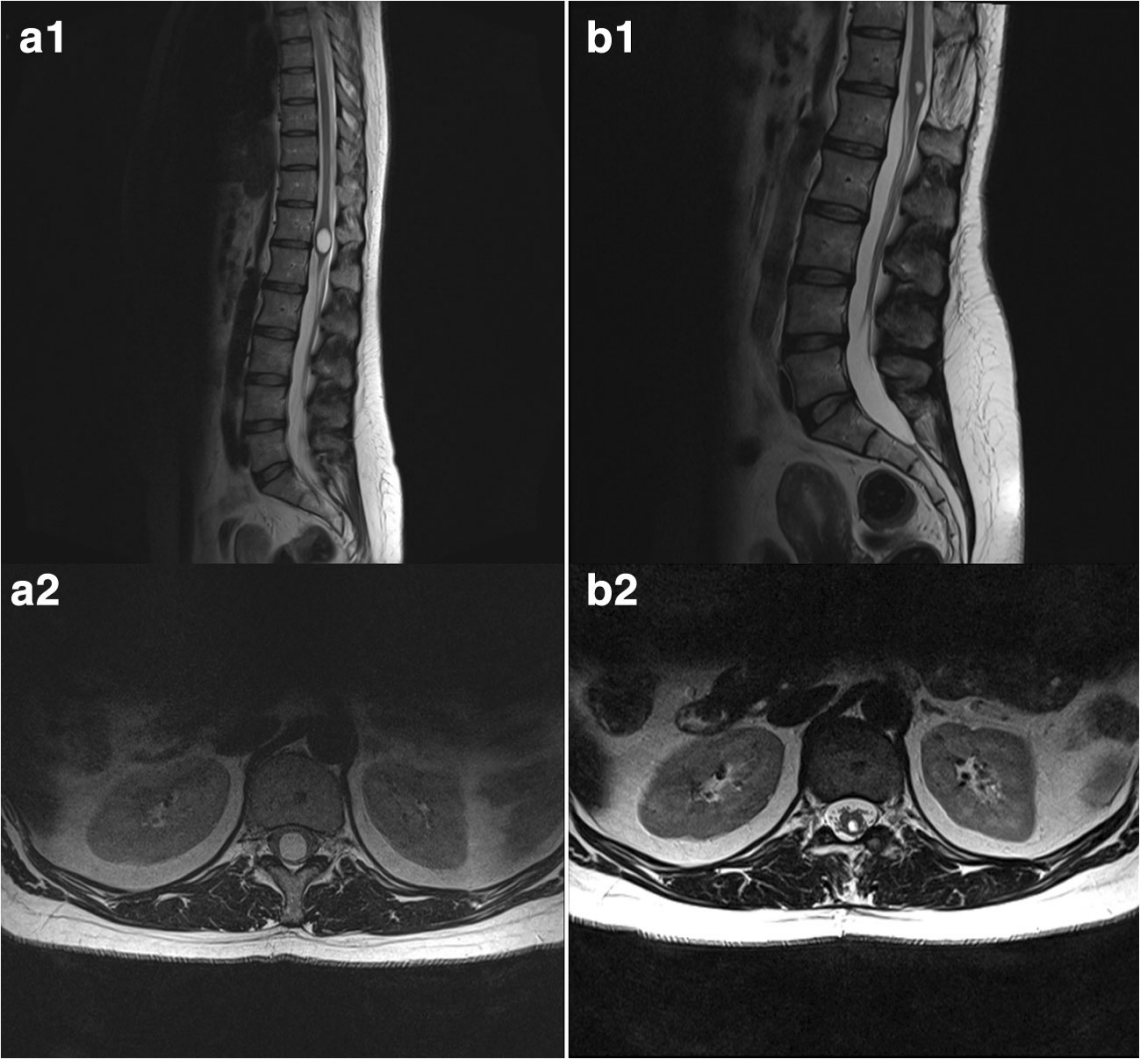
Pre-operative sagittal (A1) and axial (A2) T2-weighted magnetic resonance images showing an intramedullary ventriculus terminalis. Postoperative sagittal (B1) and axial (B2) T2-weighted magnetic resonance images showing the same lesion 3 months after cyst fenestration

**Fig. 2:**
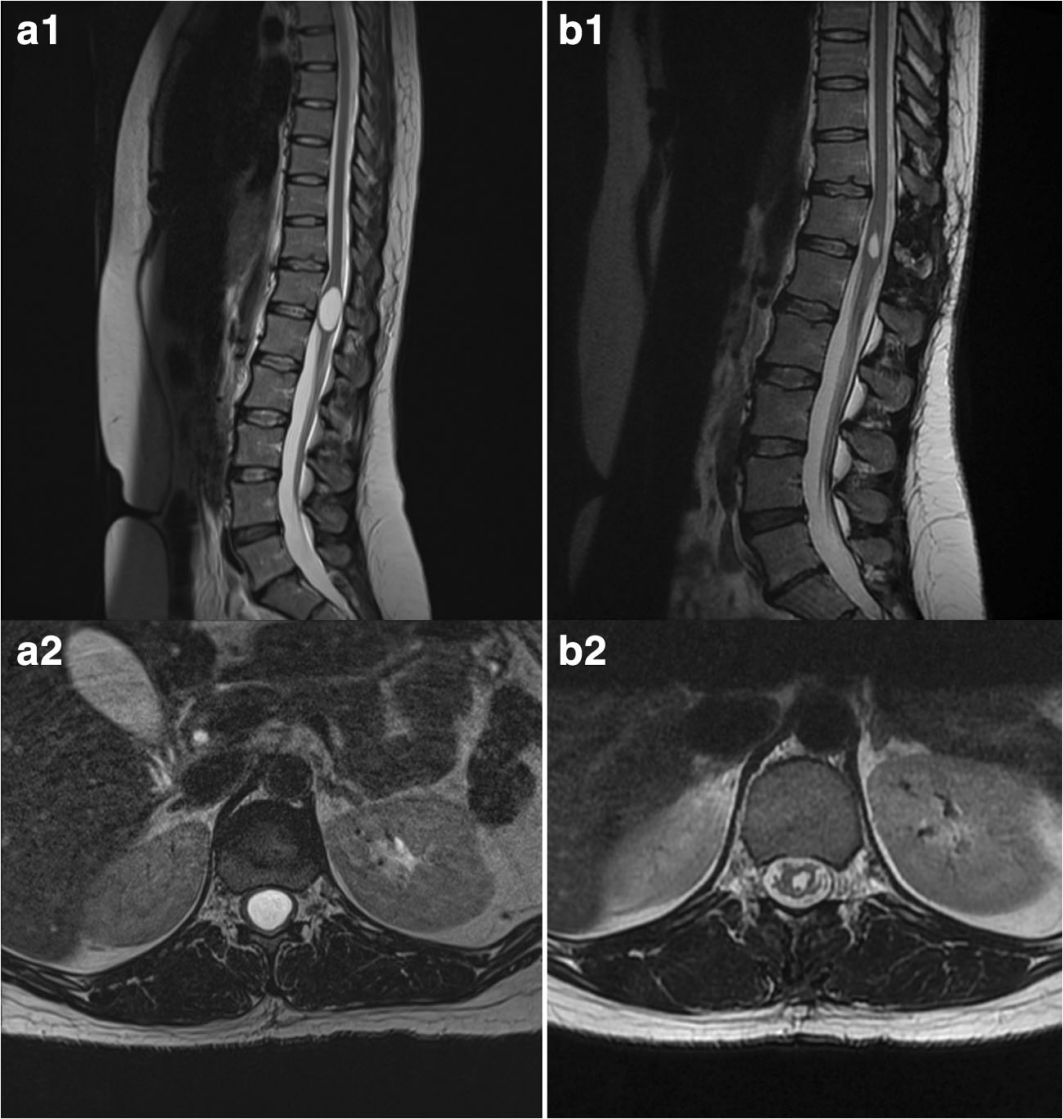
Pre-operative sagittal (A1) and axial (A2) T2-weighted magnetic resonance images showing an intramedullary ventriculus terminalis. Postoperative sagittal (B1) and axial (B2) T2-weighted magnetic resonance images showing the same lesion 3 months after midline myelotomy and placement of a cyst-subarachnoid shunt

Ventriculus terminalis, also known as the fifth ventricle, is an ependymal lined cerebrospinal fluid (CSF)-filled cavity within the conus medullaris. It is formed between the 43rd and the 48th day of embryogenesis, via canalization and retrogressive differentiation of the caudal end of the developing spinal cord and typically regresses completely after birth [1]. However, in rare cases, a residual ventricular cyst may persist into adulthood [2] (Figs. 1 and 2) and give rise to neurological symptoms, including sensorimotor disturbances and urorectal dysfunction [3]. Contrary to intramedullary tumors and/or syringomyelia, ventriculus terminalis is rounded, not contrast enhancing and exclusively found in the conus region of the spine [4].

The treatment for symptomatic ventriculus terminalis, whether conservative or surgical, remains uncertain. To date, there are only 54 described cases of surgical treatment reported in the literature [2–24] (Table 1). An important challenge is to identify patients where the potential benefits of surgery outweigh the risks. In an effort to facilitate this, de Moura Batista and colleagues established a clinical classification system based on the available literature (“cystic lesion of the ventriculus terminalis classification” (CLVT) [10], later revised by Ganau et al. based on their cohort of 13 patients [2], in which patients are categorized into CLVT type Ia (stable nonspecific symptoms without clear relation to ventriculus terminalis), type Ib (nonspecific but progressing symptoms), type II (focal neurological deficits), and type III (sphincter disturbances). The system proposes that type Ia lesions are best managed conservatively, while surgery is recommended for the remaining types.

**Table 1:** Reported cases of surgical treatment for ventriculus terminalis

Study	Patients (*n*)	Surgical method	Symptom outcome
Agrillo et al. 1997 [5]	1	Fenestration	Improved
Bellocchi et al. 2013 [6]	1	Fenestration	Improved
Borius et al. 2010 [7]	1	Fenestration	Improved
Brisman et al. 2006 [8]	1	Fenestration	Improved
Ciappetta et al. 2008 [9]	2	Fenestration	Improved
de Moura Batista et al. 2008 [10]	2	Fenestration	Improved
Dhillon et al. 2010 [11]	1	Fenestration	Improved
Dullerud et al. 2003 [12]	2	Fenestration	Improved (1), unchanged (1)
Ganau et al. 2012 [2]	10	Fenestration	Improved
Kawanishi et al. 2016 [13]	1	Fenestration and cyst-subarachnoid shunt	Improved
Korosue et al. 1981 [14]	1	Fenestration	Improved
Lotfinia and Mahdkhah 2018 [3]	3	Fenestration	Improved
Matsubayashi et al. 1998 [15]	2	Fenestration	Improved
Nassar et al. 1968 [16]	3	Fenestration	Improved
Pencovich et al. 2013 [17]	1	Fenestration	Improved
Severino and Severino 2017 [18]	1	Fenestration	Improved
Sigal et al. 1991 [19]	2	Fenestration	Unknown
Stewart et al. 1970 [20]	3	Fenestration	Improved
Suh et al. 2012 [4]	4	Fenestration	Improved (3), unchanged (1)
Takahashi et al. 2009 [21]	4	Fenestration (1) or magnetic resonance imaging-guided aspiration (3)	Improved
Woodley-Cook et al. 2016 [22]	1	Fenestration	Improved
Zeinali et al. 2017 [23]	1	Fenestration	Improved
Zhang et al. 2017 [24]	6	Fenestration and a cyst-subarachnoid shunt	Improved (5), unchanged (1)
Present study	13	Fenestration (11) and a cyst-subarachnoid shunt (2)	Improved (11), unchanged (2)
Summary	67	Fenestration (55) and a cyst-subarachnoid shunt (9) Magnetic resonance imaging-guided aspiration (3)	Improved (60), unchanged (5), unknown (2)

The aim of this study was to present our institutional experience of patients with ventriculus terminalis. In view of the limited data available in the literature, the presented material can provide additional insight to assist in decision-making for this patient category.

## Methods

All adult patients (≥ 15 years) with ventriculus terminalis who were referred to the Department of Neurosurgery, Karolinska University Hospital (Stockholm, Sweden), between 2010 and 2018, were included in the study. No patients were excluded. The Karolinska University Hospital is a publicly funded and owned tertiary care center serving a region of approximately 2 million inhabitants and the only hospital in the region that accepts referrals for ventriculus terminalis. Thus, there was no selection bias. Medical records and imaging data from digital hospital charts were retrospectively reviewed using the health record software TakeCare (CompuGroup Medical Sweden AB, Farsta, Sweden). Outcome was assessed by change in cyst size and clinical status. The study was approved by the Regional Ethical Review Board in Stockholm, Sweden (Dnr: 2016/1708-31/4) who waived the need for informed consent.

### Patient management

Following referral, a detailed neurological examination was performed in all patients. Special attention was given to any differential diagnoses that might explain the patients’ symptoms. Patients were offered surgery if they had an MRI-verified ventriculus terminalis and symptoms consistent with compression at the level of the conus medullaris.

The surgical treatment of choice was cyst fenestration through a laminotomy and subsequent myelotomy. Prior to surgery, the spinous process of the vertebra above the cyst was identified using CT guidance and marked with injection of a sterile carbon suspension. With the patient in the prone position, a laminotomy was performed via a midline approach, using an ultrasonic bone scalpel (Misonix Inc., Farmingdale, NY, USA). Under the microscope, the dura was incised in the midline and held open by sutures, after which the arachnoid was opened and the cyst exposed. Following this, a 4–5-mm longitudinal fenestration was made, using a sharp cannula or a diamond knife, at the thinnest part of the cyst wall. If there was no apparent thin area, a midline fenestration was performed instead. In two patients with no evident cyst wall, a midline fenestration was performed and a cyst-subarachnoid shunt was placed and fixed with non-absorbable sutures. The dura was then closed using resorbable sutures, and the lamina affixed with titanium microplates. No duraplasty or expansion was performed. Following surgery, patients were kept on bed rest for 24 h and subsequently mobilized. All patients were discharged to a rehabilitation facility before returning home.

In adherence with routine protocols, all surgically treated patients underwent a follow-up MRI and clinical examination by the treating physician/surgeon after 3 months. Additional MRI at other time-points was performed in selected cases, when clinically indicated. To assess long-term clinical outcome, all patients were evaluated with a telephone interview at an average (median) of 61 months (range 7–124) after surgery.

## Results

### Baseline characteristics

During the study period, 14 patients were referred for MRI-verified ventriculus terminalis. All patients were symptomatic at the time of referral, and the most common symptom was lower limb weakness (*n* = 9). Thirteen (93%) of the patients were female, and the median age was 45 years (range 35–71). The median cyst volume, as measured by a neuroradiologist using the formula (length × width × height)/2, was 2 ml (range 0.4–23). The lesions were located at levels T11–T12 (*n* = 4), T12–L1 (*n* = 9), and L2–S2 (*n* = 1). None of the patients had previously undergone spinal surgery. Eight patients had a concurrent spinal pathology (lumbo-sacral disk herniation (*n* = 4), lumbo-sacral disk degeneration (*n* = 2), combined cervical disk herniation and spinal stenosis (*n* = 1), and combined cervical spinal stenosis and a sacral perineural cyst (*n* = 1)). However, none of these concurrent pathologies were believed to adequately explain all the patients’ symptoms. All patients were retrospectively categorized based on the CLVT classification (two type Ia, one type Ib, four type II, and seven type III) (Table 2). The CLVT classification system was not used in the selection of surgical candidates in our cohort.

**Table 2:** Patients referred to the Karolinska University Hospital for symptomatic ventriculus terminalis between 2010 and 2018

Number	Age	Sex	Symptoms	Symptom duration (months)	CLVT type	Spinal segment	Concurrent spinal pathology	Treatment	Cyst volume (ml) pre-/postop	Follow-up time (months)	Long-term complication	Long-term clinical status
1	63	F	Urinary incontinence Left-sided leg weakness	9	III	T11–T12	–	Fenestration	12/1.5	103	Partial cyst recurrence, re-operation	Improved
2	50	F	Urinary incontinence Bilateral leg weakness, paresthesia, and pain	24	III	T11–T12	–	Fenestration and cyst-subarachnoid shunt	2/0.25	16	–	Improved
3	36	F	Progressing back pain	120	Ib	T11–T12	–	Fenestration	2/0.75	85	–	Improved (complete)
4	45	F	Urinary incontinence Sciatica Bilateral leg weakness and paresthesia	30	III	T12–L1	Herniated disk L5–S1	Fenestration	0.4/0.1	60	–	Improved
5	38	F	Anal sphincter dysfunction Sciatica	24	III	T12–L1	Herniated disk L5–S1	Fenestration	2/0.1	61	–	Improved
6	44	F	Detrusor hypofunction Bilateral leg weakness and paresthesia	12	III	T12–L1	Disk degeneration L4–L5 and L5–S1	Fenestration	9/2	99	Partial cyst recurrence, no re-operation	Improved
7	42	F	Bilateral leg paresthesia	12	II	T12–L1	–	Fenestration	1/0.04	90	Partial cyst recurrence, no re-operation	Unchanged
8	53	F	Right-sided leg weakness and pain	18	II	T12–L1	Herniated disk C4–C5 Spinal stenosis C5–C6	Fenestration	1/ 0.2	96	–	Improved
9	71	M	Detrusor hypofunction Bilateral leg weakness	18	III	T12–L1	–	Fenestration	5/0.5	59	–	Improved
10	64	F	Urinary incontinence Anal sphincter dysfunction Lower back pain Bilateral leg weakness and paresthesia	60	III	L2–S2	Spinal stenosis C5–C6 Synovial cyst S3–S4	Fenestration of ventriculus terminalis and perineural cyst	23/0.01	15	–	Improved
11	40	F	Sciatica (non-progressing)	60	Ia	T12–L1	Disk degeneration L4–L5 and L5–S1	Fenestration and cyst-subarachnoid shunt	3/0.15	7	–	Unchanged
12	35	F	Bilateral leg weakness Sciatica	30	II	T11–T12	Herniated disk L5–S1	Fenestration	1/0.05	11	–	Improved
13	56	F	Right-sided leg weakness Sciatica	24	II	T12–L1	Disk degeneration L4–L5	Fenestration	4/0.1	16	–	Improved
14	38	F	Sciatica (non-progressing)	12	Ia	T12–L1	–	No surgery	1.5/N/A	124	–	Deteriorated

*CLVT* cystic lesion of the ventriculus terminalis, *F* female, *M* male, *MRI* magnetic resonance imaging

### Treatment

All 14 patients were offered surgery. One patient opted for conservative treatment instead (patient no. 14), while the remaining thirteen patients underwent microsurgical cyst fenestration. The median time between the onset of symptoms and surgery was 24 months (range 9–120). The postoperative MRI confirmed cyst size reduction in all patients (Figs. 1 and 2). No postoperative complications, including pseudomeningocele, were observed.

### Outcome

For the surgically treated cohort, the median long-term follow-up time was 60 months (range 7–103). This included data acquired from patients’ primary health care providers as well as the structured telephone interview. In total, 11 (85%) of the surgically treated patients showed clinical improvement at long-term follow-up, while the other two remained unchanged. Of the 11 patients with clinical improvement, one patient experienced complete symptom relief following surgery (patient no. 3). For the remaining patients with partial symptom relief, there was no clear indication as to which specific neurological symptom was most likely to be resolved following surgery (Table 3).

**Table 3:** Detailed pre- and postoperative status for patients referred for symptomatic ventriculus terminalis between 2010 and 2018

Number	Pre-operative clinical status	Treatment	Postoperative change in clinical status
1	Urinary incontinence Left-sided leg weakness	Fenestration	Unchanged bladder function Partially improved leg weakness
2	Urinary incontinence Bilateral leg weakness, paresthesia, and pain	Fenestration and cyst-subarachnoid shunt	Completely improved bladder function Completely improved leg weakness and pain Partially improved leg paresthesia
3	Progressing back pain	Fenestration	Completely improved back pain
4	Urinary incontinence Sciatica Bilateral leg weakness and paresthesia	Fenestration	Unchanged bladder function Partially improved sciatica Unchanged leg weakness and paresthesia
5	Anal sphincter dysfunction Sciatica	Fenestration	Partially improved anal sphincter function Partially improved sciatica
6	Detrusor hypofunction Bilateral leg weakness and paresthesia	Fenestration	Partially improved bladder function Partially improved leg weakness Completely improved leg paresthesia
7	Bilateral leg paresthesia	Fenestration	Unchanged
8	Right-sided leg weakness and pain	Fenestration	Completely improved leg weakness Unchanged leg pain
9	Detrusor hypofunction Bilateral leg weakness	Fenestration	Partially improved bladder function Partially improved leg weakness
10	Urinary incontinence Anal sphincter dysfunction Lower back pain Bilateral leg weakness and paresthesia	Fenestration of ventriculus terminalis and perineural cyst	Completely improved leg weakness and paresthesia Completely improved lower back pain Unchanged bladder function Unchanged anal sphincter function
11	Sciatica (non-progressing)	Fenestration and cyst-subarachnoid shunt	Unchanged
12	Bilateral leg weakness Sciatica	Fenestration	Partially improved leg weakness Partially improved sciatica
13	Right-sided leg weakness Sciatica	Fenestration	Partially improved leg weakness Partially improved sciatica
14	Sciatica (non-progressing)	No surgery	Unchanged

Seven patients underwent additional imaging (median 44 months after surgery, range 17–89), among which partial cyst recurrence was evident in three. One of the patients with partial cyst recurrence also had concurrent symptom reccurence and thus underwent successful renewed surgical fenestration 7 years after the initial operation (patient no. 1; Table 2).

The conservatively treated patient (patient no. 14) was followed clinically and radiologically for 124 months. During follow-up, the cyst volume increased from 1.5 to 3.2 ml. Clinically, the patient’s sciatica progressed. Despite the new findings and symptoms, representing a transition from CLVT types Ia to Ib, the patient continued to decline surgical treatment.

## Discussion

Ventriculus terminalis is a rare condition typically identified in patients investigated for low back pain or neurological symptoms in the lower extremities, including urorectal symptoms [3]. The majority of the patients are female [23], although the explanation for this is unknown. Data to support evidence-based treatment guidelines is scarce. The CLVT classification system [2, 10] was designed to avoid unwarranted surgery. According to the CLVT classification, patients with type Ia lesions should be treated conservatively. This is mainly supported by case studies that have shown a stable clinical status in conservatively treated type Ia patients [2, 4]. To the best of our knowledge, there are only three previously reported cases of attempted surgical treatment for patients with type Ia lesions, all of whom were treated without surgical complications or neurological deterioration [12, 24]. However, Brisman et al. reported a case of a conservatively treated type Ia patient who developed acute cauda equina syndrome and required emergency surgery, highlighting the fact that these patients may deteriorate due to cyst growth [8]. Similarly, the type Ia patient in our cohort, who opted to be treated conservatively (patient no. 14), showed progressing cyst size and clinical deterioration at long-term follow-up. Considering the risk of cyst growth, as well as the fact that cyst fenestration for ventriculus terminalis appears to be safe and effective, we argue that surgery should be offered for type Ia lesions as well.

Despite MRI-verified cyst size reduction, two of our patients experienced no symptom relief following surgery (patient no. 7 and 11). One reason behind the dissociation between radiological and clinical outcome may be the prevalence of concurrent spinal pathology, making it difficult to distinguish symptoms related to the ventriculus terminalis from those related to the degenerative disease. This is further illustrated by the global incidence of asymptomatic degenerative spinal disorders estimated to be as high as 19–84% [25]. Considering this, exhaustive diagnostic workup is warranted, but not necessarily enough to resolve this issue. In our surgically treated cohort, eight patients (62%) had concurrent spinal pathology, and all but one showed postoperative clinical improvement at long-term follow-up. Thus, we believe that excluding patients from being surgically treated, solely on the basis of concurrent spinal pathology, could result in inadequate treatment.

### Surgical method

All operated patients in our cohort were treated with laminotomy and cyst fenestration, which is the most commonly described technique [2–24]. We found that 85% of surgically treated patients sustained symptomatic improvement at long-term follow-up, which is comparable to the 87% success rate reported in the literature (Table 1). Furthermore, none of the patients developed complications that could be attributed to the surgical procedure. Thus, on a group level, surgical treatment for ventriculus terminalis appears to be both safe and effective.

Two of our surgically treated patients were treated with combined cyst fenestration and placement of a cyst-subarachnoid shunt, both without complications (patient no. 2 and 11). In theory, the shunt eliminates concerns for closure of the cyst wall and reduces the risk of postoperative cyst recurrence. This method has been reported in seven previous cases (Table 1), and there have been no reports of shunt obstruction or postoperative cyst recurrence in these patients [13, 24]. Of note, three other patients in our cohort, who did not receive cyst-subarachnoid shunts, later presented with partial cyst recurrence patients no. 1, 6 and 7. One of these patients underwent renewed surgical fenestration due to symptom reccurence (patient no 1). It is possible that this could have been avoided if a cyst-subarachnoid shunt had been placed. Thus, placement of a cyst-subarachnoid shunt appears to be safe and may decrease the risk of postoperative cyst recurrence. However, more long-term outcome data is needed to establish evidence-based treatment guidelines.

While laminotomy and cyst fenestration is the most commonly described technique for treating ventriculus terminalis, an alternative method of percutaneous aspiration using real-time MRI has been described in three patients with good results [21]. This minimally invasive method might have the advantage of shorter hospital stay and no postoperative surgical site symptoms, but has yet to be validated by others.

### Recommendation

To the best of our knowledge, this is the largest cohort study of patients with surgically treated ventriculus terminalis. Based on our experience, and in accordance with the CLVT classification, we agree that surgical treatment should be offered for type Ib, II, and III lesions to partially or completely relieve symptoms. In addition, we argue that patients with type Ia lesions should be offered surgery to decrease the risk of cyst growth and associated neurological deterioration. Furthermore, patients that decline surgery should be closely monitored as cyst growth and symptom progression may occur rapidly.

## Conclusions

Microsurgical cyst fenestration is a safe and effective treatment option for ventriculus terminalis and should be offered to all symptomatic patients.

## Abbreviations

CLVT: Cystic lesion of the ventriculus terminalis classification
CSF: Cerebrospinal fluid
CT: Computed tomography
MRI: Magnetic resonance imaging

## References

[1] Van Rillaer O, Vandaele P, Ramboer K Malformative persistence of terminal ventricle. JBR-BTR: organe de la Societe royale belge de radiologie (SRBR) = orgaan van de Koninklijke Belgische Vereniging voor Radiologie (KBVR) 92(3):178–179 19670587

[2] Ganau M, Talacchi A, Cecchi PC, Ghimenton C, Gerosa M, Faccioli F (2012). Cystic dilation of the ventriculus terminalis. J Neurosurg Spine.

[3] Lotfinia I, Mahdkhah A (2018). The cystic dilation of ventriculus terminalis with neurological symptoms: three case reports and a literature review. J Spinal Cord Med.

[4] Suh SH, Chung T-S, Lee S-K, Cho Y-E, Kim KS (2012). Ventriculus terminalis in adults: unusual magnetic resonance imaging features and review of the literature. Korean J Radiol.

[5] Agrillo U, Tirendi MN, Nardi PV (1997). Symptomatic cystic dilatation of V ventricle: case report and review of the literature. Eur Spine J: official publication of the European Spine Society, the European Spinal Deformity Society, and the European Section of the Cervical Spine Research Society.

[6] Bellocchi S, Vidale S, Casiraghi P, Arnaboldi M, Taborelli A (2013). Multilobed cystic dilaetion of the ventriculus terminalis (CDVT). BMJ Case Rep.

[7] Borius P-Y, Cintas P, Lagarrigue J (2010). Ventriculus terminalis dilatation in adults: a case report and review of the literature. Neuro-Chirurgie.

[8] Brisman JL, Li M, Hamilton D, Mayberg MR, Newell DW (2006). Cystic dilation of the conus ventriculus terminalis presenting as an acute cauda equina syndrome relieved by decompression and cyst drainage: case report. Neurosurgery.

[9] Ciappetta P, D’urso PI, Luzzi S, Ingravallo G, Cimmino A, Resta L (2008). Cystic dilation of the ventriculus terminalis in adults. J Neurosurg Spine.

[10] De Moura Batista L, Acioly MA, Carvalho CH, Ebner FH, Tatagiba M (2008). Cystic lesion of the ventriculus terminalis: proposal for a new clinical classification. J Neurosurg Spine.

[11] Dhillon RS, McKelvie PA, Wang YY, Han T, Murphy M (2010). Cystic lesion of the ventriculus terminalis in an adult. J Clin Neurosci: official journal of the Neurosurgical Society of Australasia.

[12] Dullerud R, Server A, Berg-Johnsen J (2003). MR imaging of ventriculus terminalis of the conus medullaris. A report of two operated patients and a review of the literature. Acta Radiol (Stockholm, Sweden: 1987).

[13] Kawanishi M, Tanaka H, Yokoyama K, Yamada M (2016). Cystic dilation of the ventriculus terminalis. J Neurosci Rural Pract.

[14] Korosue K, Shibasaki H, Kuroiwa Y, Machi T, Sawada K, Kitamura K, Ikeda J (1981). Cyst of the conus medullaris manifesting amyotrophic lateral sclerosis syndrome. Folia Psychiatr Neurol Jpn.

[15] Matsubayashi R, Uchino A, Kato A, Kudo S, Sakai S, Murata S (1998). Cystic dilatation of ventriculus terminalis in adults: MRI. Neuroradiology.

[16] Nassar SI, Correll JW, Housepian EM (1968). Intramedullary cystic lesions of the conus medullaris. J Neurol Neurosurg Psychiatry.

[17] Pencovich N, Ben-Sira L, Constantini S (2013). Massive cystic dilatation within a tethered filum terminale causing cauda equina compression and mimicking syringomyelia in a young adult patient. Childs Nerv Syst: ChNS: official journal of the International Society for Pediatric Neurosurgery.

[18] Severino R, Severino P (2017). Surgery or not? A case of ventriculus terminalis in an adult patient. J Spine Surg (Hong Kong).

[19] Sigal R, Denys A, Halimi P, Shapeero L, Doyon D, Boudghène F Ventriculus terminalis of the conus medullaris: MR imaging in four patients with congenital dilatation. AJNR Am J Neuroradiol 12(4):733–737 PMC8331601 1882755

[20] Stewart DH, King RB, Lourie H (1970). Surgical drainage of cyst of the conus medullaris. J Neurosurg.

[21] Takahashi S, Saruhashi Y, Odate S, Matsusue Y, Morikawa S (2009). Percutaneous aspiration of spinal terminal ventricle cysts using real-time magnetic resonance imaging and navigation. Spine.

[22] Woodley-Cook J, Konieczny M, Spears J (2016). The slowly enlarging ventriculus terminalis. Pol J Radiol.

[23] Zeinali M, Safari H, Rasras S, Bahrami R, Arjipour M, Ostadrahimi N (2017). Cystic dilation of a ventriculus terminalis. Case report and review of the literature. Br J Neurosurg.

[24] Zhang L, Zhang Z, Yang W, Jia W, Xu Y, Yang J (2017). Cystic dilation of the ventriculus terminalis: report of 6 surgical cases treated with cyst-subarachnoid shunting using a T-catheter. World Neurosurg.

[25] Brinjikji W, Luetmer PH, Comstock B (2015). Systematic literature review of imaging features of spinal degeneration in asymptomatic populations. AJNR Am J Neuroradiol.

